# Regulatory mechanisms and interactions of mitophagy and ketone body metabolism in cardiac metabolism

**DOI:** 10.3389/fcvm.2026.1925684

**Published:** 2026-09-01

**Authors:** Shujun Ding, Yuna Hu, Qin Zhang, Xinyi Liu, Xuan Zhao, Yajing Zhao, Wen Zhang

**Affiliations:** Fuwai Yunnan Hospital, Chinese Academy of Medical Sciences, Affiliated Cardiovascular Hospital of Kunming Medical University, Kunming, Yunnan, China

**Keywords:** ketone body, metabolic flexibility, mitochondria, mitophagy, myocardial metabolism

## Abstract

Cardiac function depends on tightly regulated energy metabolism, with mitochondria serving as key sites of cellular energy production. Mitophagy, a selective form of autophagy that maintains mitochondrial quality, has received growing attention for its role in cardiomyocyte metabolism. Under specific physiological and pathological conditions, the myocardium increases its use of ketone bodies as energy substrates, and ketone body metabolism is closely linked to mitochondrial function. However, the relationship between mitophagy and ketone body metabolism is incompletely understood, particularly in cardiovascular disease. This review summarizes their roles and regulatory mechanisms in the myocardium and evaluates evidence for a potential bidirectional relationship. Mitophagy may preserve the mitochondrial capacity required for ketone body oxidation, whereas ketone body metabolism and *β*-hydroxybutyrate-mediated signaling may regulate mitophagy and mitochondrial stress resilience. By integrating these interactions across cardiovascular disease phenotypes, this review highlights their potential therapeutic relevance and identifies priorities for mechanism-based intervention and clinical translation.

## 1 Introduction

This review examines the potential bidirectional relationship between mitophagy and ketone body metabolism in the heart. The central concept is that mitophagy may preserve the mitochondrial integrity required for efficient ketone body oxidation, whereas ketone bodies, particularly *β*-hydroxybutyrate (*β*-OHB), may in turn regulate mitophagy and strengthen mitochondrial resistance to stress. By integrating these two directions within a single framework, this review aims to clarify how mitophagy and ketone body metabolism may interact during cardiovascular disease and how this interaction could inform therapeutic strategies.

The heart relies on mitochondrial oxidative metabolism and substrate flexibility to meet its continuous energy demands (1, 2). Under normal conditions, fatty acids (FAs) and glucose provide most of the energy required by the myocardium (3, 4). During fasting, heart failure, ischemia, and other stress states, myocardial ketone body availability and utilization may increase (5, 6). Ketone bodies can serve as oxidative substrates, but they also exert signaling effects that influence redox balance, inflammation, gene expression, and mitochondrial function (7). At the same time, mitophagy selectively removes damaged mitochondria and helps preserve respiratory-chain activity, tricarboxylic acid cycle (TCA cycle) function, and metabolic adaptability (8, 9). Because ketone body oxidation occurs within mitochondria, impaired mitochondrial quality control may limit the energetic benefit of an increased ketone body supply.

Emerging evidence indicates that mitophagy may influence the myocardial capacity for ketone body oxidation by preserving mitochondrial quality (10, 11). Conversely, ketone body-based interventions may modulate mitophagy and enhance mitochondrial resilience to stress (5, 12, 13). However, the evidence for the two directions is not equally strong. Preclinical evidence that ketone bodies regulate mitophagy is increasing, whereas direct evidence that mitophagic flux determines myocardial ketone oxidation remains limited. Previous reviews have generally discussed cardiac ketone body metabolism and mitophagy as separate topics. The present review goes beyond these parallel perspectives by evaluating their potential reciprocal regulation, distinguishing relatively well-supported mechanisms from indirect or proposed links, and comparing this interaction across cardiovascular disease phenotypes and stages. It also considers whether therapeutic modulation should target ketone body availability, ketone body utilization, mitophagic flux, or a combination of these processes.

The review is organized as follows. Sections 2–5 establish the mechanistic foundation by summarizing cardiac energy metabolism, mitochondrial regulation, mitophagy, and ketone body metabolism. Section 6 integrates current evidence for their potential bidirectional interaction and compares its characteristics across cardiovascular diseases. Section 7 discusses biomarkers, therapeutic interventions, and major challenges for clinical translation. Section 8 summarizes the main conclusions and research priorities.

## 2 Overview of cardiac energy metabolism

**Figure 1:**
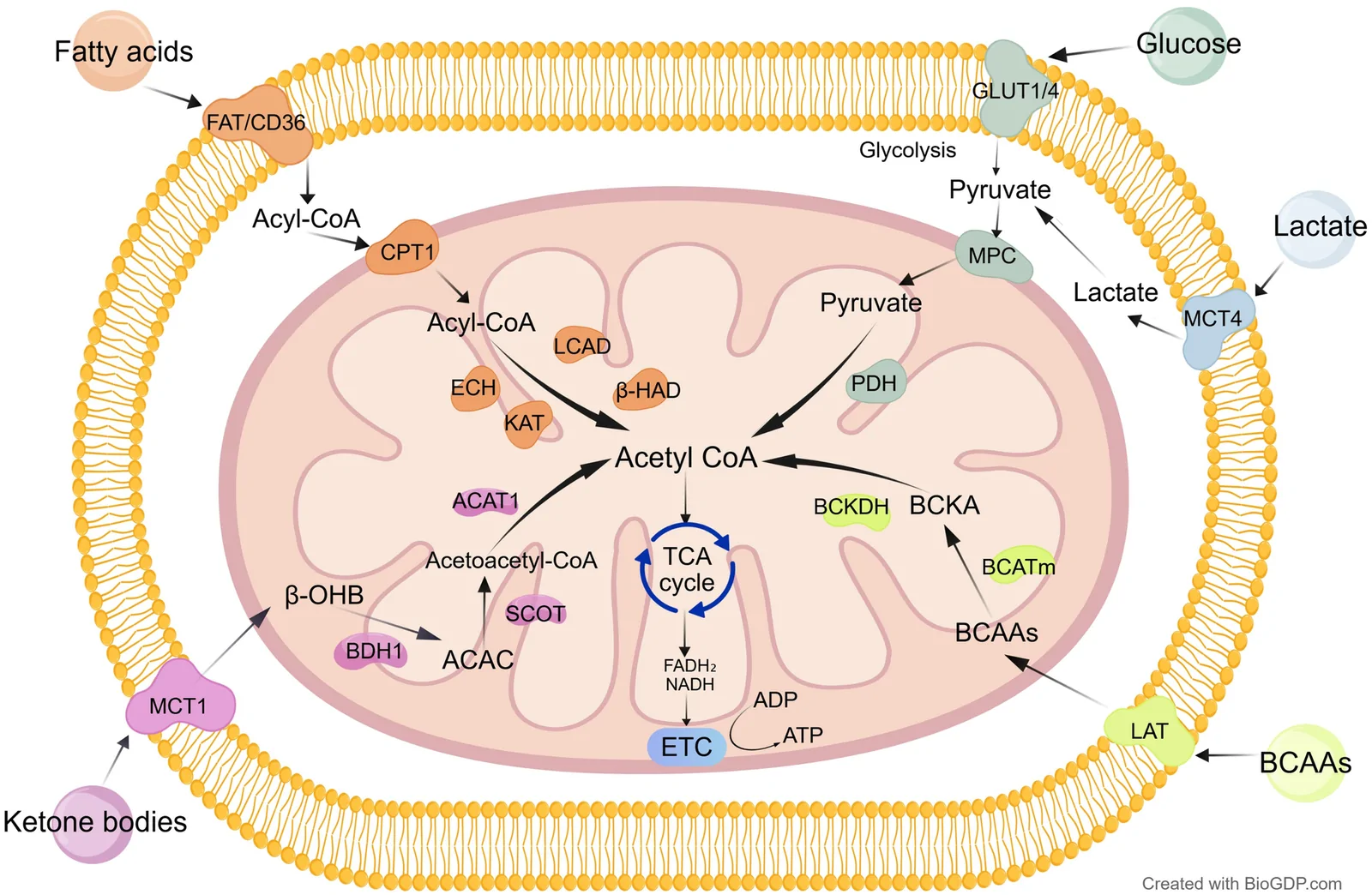
Major metabolic substrates, pathways and related Key enzymes. FAT/CD36, fatty acid translocase/cluster of differentiation 36; Acyl-CoA, acyl-coenzyme A; CPT1, carnitine palmitoyltransferase 1; ECH, enoyl-CoA hydratase; LCAD, long-chain acyl-CoA dehydrogenase; KAT, 3-ketoacyl-CoA thiolase; *β*-HAD, 3-OH-acyl-CoA dehydrogenase; GLUT1, insulin-independent glucose transporter 1; GLUT4, insulin-dependent glucose transporter 4; MPC, mitochondrial pyruvate carrier; PDH, pyruvate dehydrogenase; MCT4, monocarboxylate transporter 4; MCT1, monocarboxylate transporter 1; *β*-OHB, *β*-hydroxybutyrate; BDH1, *β*-hydroxybutyrate dehydrogenase 1; ACAC, acetoacetate; SCOT, succinyl-CoA:3-oxoacid-CoA transferase; ACAT1, acetyl-CoA acetyltransferase 1; BCAAs, branched-chain amino acids; LAT, L-type amino acid transporter; BCATm, mitochondrial branched-chain amino acid aminotransferase; BCKDH, branched-chain *α*-keto acid dehydrogenase; TCA cycle, tricarboxylic acid cycle; FADH_2_, reduced flavin adenine dinucleotide; NADH, reduced nicotinamide adenine dinucleotide; ETC, electron transport chain; ADP, adenosine diphosphate; ATP, adenosine triphosphate.

The adult heart exhibits high oxidative metabolic capacity and remarkable substrate flexibility, enabling cardiomyocytes to utilize a diverse range of fuels, including FAs, glucose, ketone bodies, and amino acids (Figure 1) (14–16). Under different conditions, cardiomyocytes dynamically adjust substrate utilization to optimize adenosine triphosphate (ATP) production and oxygen efficiency, thereby enabling the heart to maintain energy homeostasis (15, 17).

### 2.1 Metabolic substrates and metabolic pathways

#### 2.1.1 Fatty acids

Fatty acid oxidation (FAO) supplies most of the energy required by the heart (14, 15). Because FAs cannot be synthesized in the heart, myocardial FAs are derived primarily from circulating FAs released by adipose tissue, the hydrolysis of endogenous cardiac triglycerides (TGs), and the hydrolysis of circulating TG-rich lipoproteins mediated by lipoprotein lipase (LPL) (18). Circulating free fatty acids (FFAs) are taken up by cardiomyocytes through transport proteins such as fatty acid translocase/cluster of differentiation 36 (FAT/CD36) and are subsequently converted to acyl-coenzyme A (acyl-CoA) in the cytosol (19, 20). Short-chain fatty acids (SCFAs) can enter mitochondria directly, whereas long-chain fatty acids (LCFAs) require the assistance of the carnitine palmitoyltransferase system (21, 22). Acyl-CoA undergoes *β*-oxidation in the mitochondrial matrix to generate acetyl-coenzyme A (acetyl-CoA), reduced nicotinamide adenine dinucleotide (NADH), and reduced flavin adenine dinucleotide (FADH_2_) (21, 23). Acetyl-CoA then enters the TCA cycle, leading to the further production of reducing equivalents (20, 21, 24).

#### 2.1.2 Glucose

Glucose is an important energy substrate for the heart and represents one of its major fuel sources (14, 15). Glucose uptake by cardiomyocytes is determined primarily by the transmembrane glucose concentration gradient as well as the number of glucose transporters at the cell membrane, and it can be inhibited by FAs (1). Insulin-dependent glucose transporter 4 (GLUT4) is the main glucose transporter in the adult heart, whereas insulin-independent glucose transporter 1 (GLUT1) is expressed at higher levels in the fetal and neonatal heart (25, 26). Circulating glucose is taken up by cardiomyocytes through these transporters and is subsequently metabolized through cytosolic glycolysis to generate pyruvate, ATP, and NADH (15, 27). Pyruvate is then transported into mitochondria by the mitochondrial pyruvate carrier (MPC) and converted to acetyl-CoA by the pyruvate dehydrogenase (PDH) before entering the TCA cycle for further oxidation (15, 25, 27).

#### 2.1.3 Lactate

Lactate is increasingly recognized as an important energy substrate for the heart. During both rest and exercise, lactate released by muscle can be transported through the circulation and taken up by cardiomyocytes (28). In the healthy human heart, the consumption of lactate is closely matched by its simultaneous production through glycolysis (29), allowing glucose to enter mitochondrial oxidative metabolism indirectly via circulating lactate (30). After entering cardiomyocytes, lactate is converted to pyruvate by lactate dehydrogenase (LDH) (31). Pyruvate is then transported into mitochondria through the MPC, converted to acetyl-CoA and oxidized through the TCA cycle to produce ATP (29, 32). Beyond its role as a metabolic substrate, lactate may also regulate cellular processes through lactylation-mediated epigenetic modifications.

#### 2.1.4 Ketone bodies

Ketone bodies normally make a relatively limited contribution to myocardial energy production but become increasingly important under specific physiological and pathological conditions. The principal ketone bodies utilized by cardiomyocytes are *β*-OHB and its derivative acetoacetate (ACAC) (5, 14, 33). Circulating *β*-OHB is transported into cardiomyocytes through monocarboxylate transporter 1 (MCT1) and converted to ACAC in the mitochondria by *β*-hydroxybutyrate dehydrogenase 1 (BDH1) (5, 34). ACAC is subsequently converted to acetoacetyl-CoA by succinyl-CoA:3-oxoacid-CoA transferase (SCOT) and then cleaved by acetyl-CoA acetyltransferase 1 (ACAT1) to generate acetyl-CoA, which enters the TCA cycle for ATP production (32, 35).

#### 2.1.5 Amino acids

The heart can also obtain energy from amino acid metabolism, particularly from branched-chain amino acids (BCAAs) (14, 32). BCAAs are taken up through L-type amino acid transporter (LAT) and transaminated by mitochondrial branched-chain amino acid aminotransferase (BCATm) to form branched-chain ketoacids (BCKAs) (36). BCKAs are subsequently oxidatively decarboxylated by the branched-chain *α*-keto acid dehydrogenase (BCKDH) complex, generating acetyl-CoA and other intermediates that enter the TCA cycle (32, 37). Although BCAAs make only a modest direct contribution to myocardial ATP production, their circulating levels and metabolism may substantially influence cardiovascular metabolic homeostasis by modulating key signaling pathways and nitrogen balance (36).

### 2.2 Cardiac metabolic flexibility in health and disease

The healthy adult heart exhibits substantial metabolic flexibility and dynamically adjusts substrate utilization according to substrate availability, workload, hormonal status, and oxygen supply (32). FAs and glucose constitute the major energy substrates, whereas lactate, ketone bodies, and amino acids make variable contributions under specific physiological conditions, including exercise, fasting, and pregnancy. This flexibility enables the myocardium to maintain ATP production while adapting to changes in systemic metabolism and cardiac energy demand (33, 35, 38–41).

Under pathological conditions, this metabolic flexibility is often compromised, resulting in distinct patterns of substrate utilization that vary across different cardiac diseases. For example, heart failure with reduced ejection fraction (HFrEF) is typically characterized by diminished FAO and a compensatory increase in ketone body utilization (17, 42), whereas other conditions such as heart failure with preserved ejection fraction (HFpEF), diabetic cardiomyopathy, pressure-overload hypertrophy, and atrial remodeling display more heterogeneous and sometimes divergent metabolic profiles (38, 43, 44). These variations are shaped by factors including disease etiology, progression stage, cardiac region, and substrate availability. Importantly, such heterogeneity may influence whether alterations in mitophagy and ketone body metabolism serve adaptive, insufficient, or maladaptive roles in specific disease contexts. A more detailed discussion of these disease-specific metabolic phenotypes and their implications for mitophagy-ketone body interactions will be provided in section 6.

## 3 Mitochondrial control of cardiac energy metabolism

Mitochondria are the main sites of ATP production in cardiomyocytes and act as central hubs for integrating the metabolism of FAs, glucose, lactate, ketone bodies, and amino acids (45). These substrates are ultimately converted into acetyl-CoA or other intermediates of the TCA cycle, generating reducing equivalents that drive oxidative phosphorylation (46). Beyond energy production, mitochondria also regulate redox balance, calcium homeostasis, reactive oxygen species (ROS) generation, and metabolic signaling (47, 48). Thus, maintaining mitochondrial integrity and function is essential for sustaining cardiac contractility and metabolic flexibility.

When mitochondrial function is impaired, oxidative phosphorylation declines, redox balance is disrupted, and ROS accumulate (32). These changes limit the heart's ability to adjust substrate utilization in response to energy demands. Because ketone body oxidation depends on mitochondrial enzymes, an intact TCA cycle, and a functional respiratory chain (49), mitochondrial impairment may reduce the efficiency of converting ketone bodies into ATP. Importantly, these metabolic alterations differ depending on disease type, stage, cardiac region, and substrate supply, and should not be generalized across all cardiac conditions (50–52). To maintain a healthy mitochondrial network, cardiomyocytes depend on coordinated quality-control processes, including mitochondrial fission and fusion, biogenesis, and mitophagy (53). Mitophagy can selectively remove damaged mitochondria, which may contribute to preserving oxidative capacity, limiting oxidative stress, and supporting metabolic adaptability (54). However, both insufficient and excessive mitophagy, as well as impaired mitophagic flux, can disrupt energy balance (55, 56). These considerations provide a basis for understanding how mitophagy might influence myocardial ketone body utilization and how ketone body metabolism, in turn, could affect mitochondrial quality control.

## 4 Mitophagy

Autophagy is a highly conserved cellular metabolic regulatory mechanism through which cells meet their metabolic needs and renew organelles **(**57). As a form of selective autophagy, mitophagy is an important mechanism for maintaining mitochondrial quality, energy metabolism, and cellular physiological functions (58).

### 4.1 Mechanism of mitophagy

#### 4.1.1 PINK1-PRKN-mediated mitophagy

The PTEN-induced kinase 1 (PINK1)-parkin RBR E3 ubiquitin protein ligase (PRKN) pathway is the best characterized ubiquitin-dependent mitophagy mechanism. In healthy mitochondria, PINK1 is rapidly imported into the mitochondria through the translocase of the outer mitochondrial membrane/translocase of the inner mitochondrial membrane (TOM/TIM) complex and cleaved by the mitochondrial proteinase presenilin-associated rhomboid-like protein (PARL) (59). Cleaved PINK1 is subsequently retrotranslocated to the cytoplasm and degraded by the proteasome (59, 60).

When mitochondria are damaged, PINK1 import and cleavage are inhibited, resulting in its accumulates on the outer mitochondrial membrane and subsequent recruitment of the E3 ubiquitin ligase PRKN (59, 60). Activated PRKN ubiquitinates multiple outer mitochondrial membrane proteins, generating ubiquitin chains that amplify the damage signal and mark the mitochondrion for degradation (60). Autophagy receptors, including optineurin (OPTN), nuclear dot protein 52 kDa (NDP52), and TAX1 binding protein 1 (TAX1BP1), recognize these ubiquitinated mitochondrial proteins and recruit autophagy-related factors, including Unc-51-like autophagy-activating kinase 1 (ULK1), double FYVE-containing protein 1 (DFCP1), and WD repeat domain phosphoinositide-interacting protein 1 (WIPI1), to initiate phagophore formation around the damaged mitochondrion (61). The resulting mitophagosome subsequently fuses with a lysosome, allowing degradation of the enclosed mitochondrial material (62, 63).

In addition to the PINK1-PRKN-mediated pathway, other ubiquitin-mediated mechanisms may also contribute to mitophagy. For example, a PRKN-independent pathway involving the ubiquitin ligase ring finger protein 7 (RNF7) in the heart has been reported. RNF7 may directly ubiquitinate mitochondrial proteins and stabilize PINK1, thereby facilitating the clearance of damaged mitochondria (64). These findings indicate that ubiquitin-dependent mitophagy may remain active through alternative mechanisms when PRKN recruitment or activity is limited.

#### 4.1.2 Receptor-mediated mitophagy

Receptor-mediated mitophagy also contributes to mitochondrial quality control and enhances the adaptation of cardiomyocytes to metabolic stress (65, 66). FUN14 domain-containing 1 (FUNDC1), an outer mitochondrial membrane protein enriched at mitochondrial-associated endoplasmic reticulum membranes (MAMs), serves as a key mitophagy receptor and regulator of endoplasmic reticulum-mitochondrial communication (67). Through its microtubule-associated protein 1 light chain 3 (LC3)-interacting region, FUNDC1 binds LC3 and facilitates the sequestration of damaged mitochondria into autophagosomes (68). This interaction is controlled by the phosphorylation status of FUNDC1, which is dynamically regulated by upstream kinases and phosphatases (69). Both ULK1 and nuclear respiratory factor 1 (NRF-1) may regulate FUNDC1-mediated mitophagy through this pathway (70, 71). Other studies have found that Ras-related protein Rab-7a (RAB7) overexpression may promote mitophagy via a pathway dependent on the mitochondrial translation elongation factor Tu (TUFM) (72). These findings indicate that cardiac mitophagy is mediated by several partially overlapping pathways rather than by a single uniform mechanism. The relative contribution of each pathway is likely to depend on disease etiology, cardiac region, and stage of remodeling.

### 4.2 Role of mitophagy in cardiomyocytes

Cardiomyocytes are terminally differentiated cells with high mitochondrial density and constant energy demand (73). Because their regenerative capacity is limited, they rely heavily on mitochondrial quality control to maintain contractile and metabolic function (73). Dysregulation of mitophagy is commonly observed in the pathogenesis of various cardiac diseases.

In HF, an isoform shift from AMP-activated protein kinase (AMPK) catalytic subunit *α*2 (PRKAA2/AMPK *α*2) to catalytic subunit *α*1 (PRKAA1/AMPK *α*1) impairs mitophagy, leading to mitochondrial dysfunction, cardiomyocyte death and ultimately exacerbating disease progression (74). Similarly, PINK1 deficiency results in abnormal mitochondrial accumulation, structural alterations, increased oxidative stress, pathological hypertrophy, and ventricular dysfunction (75). These findings indicate that defective mitochondrial clearance contributes to cardiac remodeling and functional decline. Mitophagy may also suppress regulated cell death, including necroptosis, during remodeling (75). Mitophagy may effectively inhibit the occurrence and development of necroptosis in cardiomyocytes during cardiac remodeling (76). In myocardial infarction models, irisin enhances mitophagy by activating the dynamin-like dynamin-like guanosine triphosphatase (GTPase) optic atrophy 1 (OPA1), reduces injury and improvs cardiac function (77).

During ischemia-reperfusion injury, the role of mitophagy is more complex. Dynamin-related protein 1 (DRP1) is activated and induces mitochondrial fission, which subsequently leads to voltage-dependent anion channel 1 (VDAC1) oligomerization, the release of hexokinase 2 (HK2), the opening of the mitochondrial permeability transition pore (mPTP), the upregulation of PINK1-PRKN, and the induction of mitophagy (78). Moderate and efficient mitophagy helps remove damaged mitochondria and protect the myocardium (75, 78). In contrast, excessive clearance, impaired lysosomal degradation, or sustained activation without sufficient mitochondrial biogenesis can reduce mitochondrial capacity and worsen energy failure (56, 78–80). FUNDC1-mediated mitophagy is particularly sensitive to hypoxia and ischemia, but its effects also depend on activation timing and intensity (78, 81).

A similar phenomenon is observed in aging. With advancing age, declines in autophagosome formation, lysosomal function, and mitochondrial turnover lead to the accumulation of damaged mitochondria, increased oxidative stress, and reduced cardiac reserve (82, 83). In experimental models, AKT2 deletion can restore forkhead box O1 (FOXO1)-related autophagy, preserve mitochondrial integrity, and alleviate age-related cardiac dysfunction (84). However, interventions effective in young or healthy models may be less effective in aged hearts, where lysosomal function and mitochondrial biogenesis are already impaired (62, 85, 86).

Mitophagy represents a potential therapeutic target in cardiovascular disease, but its modulation requires careful consideration of pathway activity and flux. Importantly, increased levels of autophagy markers do not necessarily indicate enhanced mitophagic flux. Their accumulation may reflect either increased initiation or impairment of downstream processes. Therefore, mitophagy should be evaluated as a complete process, encompassing mitochondrial recognition, autophagosome formation, lysosomal delivery, and degradation. Effective mitochondrial quality control depends on maintaining a balance between mitochondrial removal and biogenesis, rather than simply increasing mitophagy activity. Therapeutic strategies should therefore aim to restore balanced mitochondrial turnover rather than indiscriminately activate mitophagy.

## 5 Ketone body metabolism

### 5.1 Synthesis and utilization of ketone bodies

Ketone bodies are synthesized primarily in the mitochondrial matrix of the liver and consist mainly of ACAC, *β*-OHB, and acetone (AC) (87). Their principal precursors are FFAs, which are released through the lipolysis of triacylglycerols in adipose tissue and transported to the liver (35). *β*-oxidation of FAs generates acetyl-CoA, which is subsequently converted to ACAC through a series of enzymatic reactions (88). Then ACAC can be reduced to *β*-OHB by BDH1, whereas a small proportion undergoes spontaneous decarboxylation to form AC (88). The rate of hepatic ketogenesis is closely associated with FAO and the availability of acetyl-CoA (87). Among the enzymes involved, 3-hydroxy-3-methylglutaryl-CoA synthase 2 (HMGCS2) is a key rate-limiting enzyme in hepatic ketone body synthesis, whereas BDH1 regulates the reversible interconversion of *β*-OHB and ACAC in both the liver and extrahepatic tissues (89). Once released into the circulation, the water-soluble ketone bodies *β*-OHB and ACAC are transported to extrahepatic tissues, including the myocardium, for energy production (88, 90). Within extrahepatic mitochondria, *β*-OHB is oxidized to ACAC by BDH1 (34, 90). ACAC is then converted to acetoacetyl-CoA via SCOT and subsequently cleaved into acetyl-CoA, which enters the TCA cycle to generate ATP **(**32). In contrast, AC contributes minimally to energy metabolism and is predominantly eliminated through exhaled air and urine (91).

### 5.2 Role of ketone bodies in cardiomyocytes

Under physiological conditions, ketone bodies make a relatively modest contribution to myocardial ATP production, but their availability and utilization increase during fasting, prolonged exercise, pregnancy, and other conditions characterized by reduced carbohydrate availability (5, 34, 92). Circulating *β*-OHB and ACAC are transported into cardiomyocytes primarily through monocarboxylate transporters and oxidized within mitochondria to generate acetyl-CoA, which enters the TCA cycle and supports oxidative phosphorylation (5, 35). Because ketone body oxidation depends on intact mitochondrial enzymes, TCA cycle activity, and respiratory-chain function, the energetic contribution of ketone bodies is determined not only by their circulating availability but also by the oxidative capacity of the myocardium.

Ketone bodies become increasingly important during cardiac stress. In the failing or hypertrophied heart, changes in FAs and glucose metabolism may be accompanied by increased circulating ketone body concentrations and altered expression of ketolytic enzymes (93, 94). In HFrEF, increased myocardial ketone body uptake and oxidation are commonly interpreted as an adaptive response to impaired conventional substrate metabolism (95). However, increased ketone body utilization does not necessarily indicate improved overall energetic efficiency, because the functional consequences depend on mitochondrial integrity, oxygen availability, disease stage, and the metabolic costs associated with the oxidation of competing substrates. Moreover, ketone body metabolic responses are not uniform across HF phenotypes, and their effects vary according to underlying metabolic status and disease characteristics.

In addition to serving as oxidative substrates, ketone bodies exert signaling effects that may influence cardiac and vascular homeostasis. *β*-OHB has been associated with modulation of oxidative stress, inflammatory signaling, gene expression, and protein post-translational modifications (96–98). It may also act through hydroxycarboxylic acid receptor 2 (HCAR2), and influence vascular, immune, and metabolic responses (35, 99). These signaling effects may occur independently of, or in parallel with, the oxidation of *β*-OHB for ATP production. Therefore, circulating ketone body concentration, myocardial ketone body uptake, mitochondrial ketone body oxidation, and *β*-OHB-mediated signaling should be treated as related but distinct biological processes. Ketone bodies may also contribute to cardiac development and mitochondrial maturation. During the neonatal transition, circulating and myocardial ketone body levels increase transiently and have been implicated in mitochondrial maturation and metabolic reprogramming of the developing heart (100).

Overall, ketone bodies act both as metabolic substrates and as signaling mediators in the myocardium. Their cardiac effects depend on systemic ketone body availability, myocardial transport and ketolytic capacity, mitochondrial function, and the underlying disease phenotype.

## 6 The interaction between mitophagy and ketone body metabolism in cardiac metabolism

### 6.1 Bidirectional interaction between mitophagy and ketone body metabolism

Mitophagy and ketone body metabolism are closely linked to mitochondrial function and cardiac metabolic adaptation. Mitophagy maintains mitochondrial quality by removing damaged or developmentally immature mitochondria. Ketone body oxidation provides acetyl-CoA for the TCA cycle, while *β*-OHB can also regulate mitochondrial stress responses through signaling mechanisms. Current findings therefore support a potential bidirectional relationship between these processes. However, the two regulatory directions are not supported equally. Experimental evidence that *β*-OHB and ketone body-based interventions affect mitophagy is stronger than evidence that mitophagy directly regulates myocardial ketone body oxidation. This imbalance may partly reflect the focus of previous interventional research. Many studies have examined ketone body supplementation and its effects on mitochondrial quality control, whereas few have manipulated mitophagy while directly measuring ketone body oxidation. The difference in evidence strength should therefore not be interpreted as proof that one regulatory direction is biologically more important than the other.

**Figure 2:**
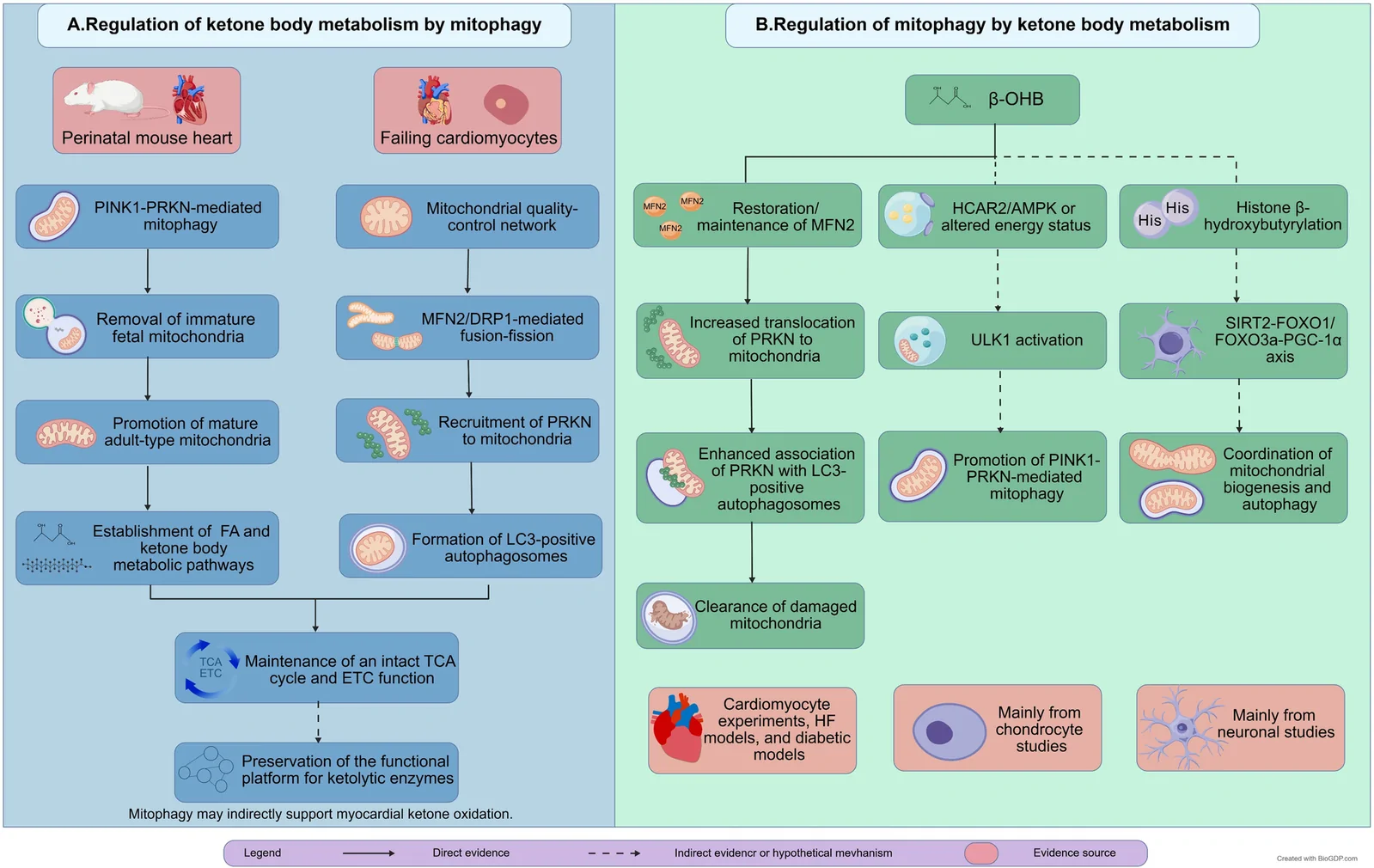
Proposed bidirectional regulatory relationship between mitophagy and ketone body metabolism. This conceptual model summarizes the potential reciprocal interaction between mitophagy and ketone body metabolism and distinguishes pathways according to the relative strength and source of the available evidence. **(A)** Regulation of ketone body metabolism by mitophagy. Mitophagy may indirectly support myocardial ketone oxidation by preserving mitochondrial quality. However, this direction is supported mainly by developmental cardiac, failing cardiomyocyte, and hepatic studies, and direct evidence linking mitophagic flux to myocardial ketone oxidation remains limited. **(B)** Regulation of mitophagy by ketone body metabolism. *β*-OHB may regulate mitophagy through several pathways. Direct evidence comes from heart-related studies, whereas indirect evidence are supported mainly by chondrocyte and neuronal studies. Solid arrows indicate relatively direct evidence, whereas dashed arrows indicate indirect or hypothetical links. PINK1, PTEN-induced kinase 1; PRKN, parkin RBR E3 ubiquitin protein ligase; FA, fatty acid; TCA cycle, tricarboxylic acid cycle; ETC, electron transport chain; MFN2, mitofusin 2; DRP1, dynamin-related protein 1; LC3, microtubule-associated protein 1 light chain 3; HF, heart failure; *β*-OHB, *β*-hydroxybutyrate; HCAR2, hydroxycarboxylic acid receptor 2; AMPK, AMP-activated protein kinase; ULK1, Unc-51-like autophagy-activating kinase 1; SIRT2, sirtuin 2; FOXO1, forkhead box O1; FOXO3a, forkhead box O3a; PGC-1*α*, peroxisome proliferator-activated receptor-*γ* coactivator 1*α*.

Figure 2 presents a conceptual model of this potential reciprocal relationship. It distinguishes relatively well-supported pathways from indirect and hypothetical links and identifies the experimental settings from which the evidence was derived. Most supporting studies are preclinical, and the proposed framework has not been fully validated in humans. Table 1 summarizes the main kinases, transcription factors, metabolic enzymes, and post-translational modifications involved in the two proposed directions.

**Table 1:** Molecular mediators of the bidirectional interaction between mitophagy and ketone body metabolism.

A. Regulation of ketone body metabolism by mitophagy			
Molecular mediator	Target or modification site	Proposed effect on ketone body metabolism	Evidence categories
PINK1	MFN2 Thr111 and Ser442 phosphorylation	Converts MFN2 into the mitochondrial PRKN receptor and promotes perinatal mitochondrial renewal, which is essential for normal ketone body metabolism	Cardiac evidence for mitochondrial maturation; indirect evidence for ketone body oxidation (101)
MFN2	Thr111 and Ser442	Links mitochondrial dynamics and PRKN recruitment to establishment of the mature oxidative metabolic phenotype	Direct cardiac genetic evidence; ketone body levels were not measured (101)
PRKN	Ubiquitination of MFN2 and other outer mitochondrial membrane proteins	Removes immature or dysfunctional mitochondria and supports establishment of mitochondria competent for ketone body metabolism	Direct evidence for mitophagy; indirect for ketone body metabolism (101)
BNIP3	LC3-interacting region	Maintains hepatic mitochondrial integrity and FAO, an upstream requirement for ketogenesis	Direct hepatic evidence; not demonstrated in cardiac ketone body metabolism (104)

B. Regulation of mitophagy by ketone body metabolism			
Molecular mediator	Target or modification site	Effect on mitophagy or mitochondrial quality control	Evidence categories
PRKN	Ser65 phosphorylation	*β*-OHB or ketone ester increases PRKN puncta, LC3 association, and mitochondrial clearance	Direct cardiomyocyte and noncardiac model evidence; dependence on ketone body oxidation remains unproven (102, 107)
MFN2	No ketone body-induced site identified	An intact MFN2-dependent fusion system is required for effective PRKN-LC3 coupling after β-OHB exposure	Direct cardiomyocyte evidence; modification mechanism unresolved (102)
DRP1	No ketone body-induced site identified	Reduced DRP1 may impair mitochondrial degradation and β-OHB-associated mitophagy	Cardiac association; causality not established (102)
SIRT2	Deacetylation targets	Mediates β-OHB-induced nuclear accumulation of FOXO1 and FOXO3a and promotes autophagy-related gene expression	Direct SIRT2-dependent evidence in neurons; cardiac relevance unconfirmed (106)
FOXO1	SIRT2-associated deacetylation; exact site not identified	Promotes expression of autophagy and mitochondrial quality control genes	Evidence in neurons; cardiac relevance unconfirmed (106)
FOXO3a	SIRT2-associated deacetylation; exact site not identified	Promotes autophagy-related and antioxidant gene expression	Evidence in neurons; cardiac relevance unconfirmed (106)
PGC-1*α*	SIRT2-dependent regulation; exact site not identified	Coordinates mitochondrial biogenesis with mitochondrial turnover	Direct mitophagy effect and cardiac relevance unconfirmed (106)
TFEB	Reduced phosphorylation at Ser142	Favors TFEB activation and lysosomal biogenesis, potentially supporting mitophagy	Directly measured in neurons; cardiac relevance unconfirmed (106)
PINK1	Phosphorylates PRKN and ubiquitin at Ser65	Initiates mitochondrial damage recognition and amplifies the mitophagy signal	Direct noncardiac evidence in the β-OHB pathway; cardiac relevance unconfirmed (107)
AMPK	Thr172 phosphorylation	Activates the PINK1-PRKN pathway and promotes mitophagy after β-OHB treatment	Directly measured in chondrocytes; cardiac relevance unconfirmed (107)
HCAR2	No established site relevant to this pathway	Acts as a β-OHB sensor upstream of AMPK and PINK1-PRKN signaling	Direct noncardiac mechanistic evidence; cardiac relevance unconfirmed (107)

#### 6.1.1 Regulation of ketone body metabolism by mitophagy

Ketone body oxidation occurs within mitochondria and requires a functionally competent mitochondrial population (33). Damage to the respiratory chain, redox systems, or TCA cycle may restrict ketone body utilization even when circulating ketone body levels are elevated. By removing dysfunctional mitochondria and coordinating with mitochondrial biogenesis, mitophagy may help preserve the oxidative capacity required for ketone body metabolism.

Gong et al. showed that PINK1-mitofusin 2 (MFN2)-PRKN-mediated mitophagy is required for the perinatal replacement of fetal mitochondria with mature mitochondria (101). Disruption of PRKN-mediated mitophagy impaired the development of adult mitochondrial morphology and respiratory function. It also impaired the postnatal induction of pathways related to FAs and ketone body metabolism (101). These findings support an important role for mitophagy in establishing the mature cardiac metabolic program. However, myocardial ketone body oxidation was not directly measured in this study. The results also did not show that mitophagy regulates BDH1 or SCOT activity. Thus, the study supports a role for mitophagy in developing mitochondria capable of ketone body utilization, rather than demonstrating that increased mitophagic flux directly enhances ketone body oxidation. Defective mitochondrial dynamics may further limit metabolic adaptation in the failing heart. Cardiomyocytes isolated from failing hearts showed reduced MFN2 and DRP1 expression, impaired PRKN recruitment, and incomplete delivery of damaged mitochondria to LC3-positive autophagosomes (102). Ketone body oxidation was not quantified. Nevertheless, these findings suggest that disruption of the mitochondrial dynamics–mitophagy network may reduce the ability of cardiomyocytes to respond to an increased ketone body supply.

Studies outside the heart also suggest that mitophagy participates in systemic ketone body homeostasis. In the liver, BCL2-interacting protein 3 (BNIP3)-dependent mitophagy regulates mitochondrial abundance, FAO, and metabolic zonation (103). Because hepatic ketogenesis depends on mitochondrial FAO, impaired mitochondrial quality control could alter ketone body production. However, the effects appear to be context-dependent. BNIP3 deficiency has been associated with reduced FAO and lipid accumulation, while increased tissue *β*-OHB levels have also been reported under some conditions (104). Such increases may reflect changes in ketone body production, utilization, export, or redox balance rather than more effective ketogenesis.

Collectively, current findings provide moderate support for the view that mitophagy preserves the mitochondrial quality required for ketone body utilization. PRKN-mediated mitophagy is important for establishing the mature cardiac metabolic program, and hepatic studies show that mitochondrial turnover affects pathways upstream of ketogenesis. However, a direct causal relationship between mitophagic flux and myocardial ketone oxidation has not been demonstrated. Studies that manipulate mitophagy while simultaneously measuring ketone uptake and oxidation are needed to test this direction directly.

#### 6.1.2 Regulation of mitophagy by ketone body metabolism

More studies have examined the effects of ketone bodies, particularly *β*-OHB, on mitophagy and mitochondrial quality control. *β*-OHB acts as both an oxidative substrate and a signaling metabolite (105). Its effects have been associated with oxidative stress, cellular energy status, AMPK signaling, sirtuin activity, protein *β*-hydroxybutyrylation, and PINK1-PRKN-mediated mitochondrial clearance (106, 107). However, it remains unclear whether these effects require *β*-OHB oxidation or arise from oxidation-independent signaling.

In isolated rabbit cardiomyocytes, *β*-OHB increased PRKN recruitment to mitochondria and promoted the association of PRKN-positive mitochondria with LC3-positive autophagosomes (102). These responses were observed in young and nonfailing aged cardiomyocytes but were impaired in cells from failing hearts (102). Restoring MFN2 improved PRKN-LC3 coupling, suggesting that the response to *β*-OHB requires an intact mitochondrial dynamics-autophagy system. These findings provide direct cardiac evidence that *β*-OHB can influence mitophagy-related processes, although mitophagic flux was not comprehensively quantified. Additional evidence comes from db/db mice with diabetic cardiomyopathy. Ketone ester supplementation restored myocardial BDH1 and SCOT expression, improved mitochondrial respiration, and prevented functional decline (108). It also increased MFN2 expression, promoted PRKN translocation to mitochondria, improved PRKN–LC3 association, and restored autophagic degradation (108). These findings show that ketone body utilization and mitochondrial quality-control indices can improve in parallel in the diabetic heart. However, the study did not establish whether *β*-OHB oxidation was required for mitophagy activation. The observed effects could have resulted from ketone body oxidation, *β*-OHB-mediated signaling, systemic metabolic changes, or a combination of these mechanisms. Parallel improvement in ketolytic proteins and mitophagy-related markers therefore does not by itself demonstrate a direct causal pathway. *β*-OHB has also shown autophagy-dependent cardioprotection during myocardial ischemia-reperfusion. Administration at reperfusion reduced infarct size, preserved mitochondrial membrane potential, lowered oxidative stress, increased autophagy, and improved cardiac function in mice (109). However, this study mainly assessed general autophagy and did not conclusively demonstrate selective mitochondrial clearance. It therefore provides indirect rather than definitive evidence for mitophagy.

Several possible mechanisms have also been identified outside the heart. In chondrocytes, *β*-OHB promoted PINK1-PRKN-mediated mitophagy through the HCAR2-AMPK pathway (107). In neuronal models, *β*-OHB regulated sirtuin 2 (SIRT2)-dependent FOXO1, forkhead box O3a (FOXO3a), and peroxisome proliferator-activated receptor-*γ* coactivator 1*α* (PGC-1*α*) signaling and promoted mitochondrial turnover and biogenesis (106). These studies show that *β*-OHB can activate mitochondrial quality-control pathways in several cell types. Whether the same mechanisms operate in cardiomyocytes under cardiovascular disease conditions remains uncertain.

Overall, available evidence more strongly supports ketone-mediated regulation of mitophagy than the reverse direction. However, this difference may partly reflect the greater number of studies testing ketone-based interventions. Most cardiac evidence remains preclinical, and no human study has directly shown that ketone administration increases myocardial mitophagic flux. Future studies should distinguish the effects of *β*-OHB oxidation from its receptor-mediated and intracellular signaling actions.

### 6.2 Metabolic phenotypes in heart diseases: ketone bodies and mitophagy

Metabolic heterogeneity contributes to differences in cardiovascular disease phenotypes. Mitochondrial quality control and substrate utilization are key components of these adaptations. As noted in Section 6.1, ketone metabolism and mitophagy may influence each other, but the evidence is uneven. This section reviews changes in ketone metabolism and mitophagy across major cardiac conditions, including heart failure, ischemic heart disease, hypertensive and hypertrophic cardiomyopathy, age-related myocardial degeneration, and atrial fibrillation. Both processes are often altered in these diseases, but this does not imply a direct causal link. The following discussion separates disease-specific observations from proposed mechanistic associations.

#### 6.2.1 HFrEF and HFpEF

HF is accompanied by extensive metabolic remodeling, but the pattern differs between HFrEF and HFpEF. These differences reflect distinct disease mechanisms and affect substrate utilization, mitochondrial function, and mitochondrial quality control.

##### 6.2.1.1 HFrEF

In HFrEF, the failing myocardium exhibits a marked decline in mitochondrial oxidative capacity. FAO is reduced because key metabolic pathways are downregulated (17, 38). Although glycolysis often increases, glucose oxidation does not rise proportionally (110). This mismatch reduces the efficiency of ATP production and promotes the accumulation of metabolic intermediates.

As myocardial energy production declines, the failing heart relies more on alternative substrates, particularly ketone bodies. Experimental and human studies have consistently reported higher circulating ketone body levels, increased myocardial ketone body uptake, and upregulation of ketolytic enzymes (94, 95, 111). These changes are generally considered an adaptive response that may partly compensate for impaired oxidation of FAs and glucose. Mitophagy also changes during the progression of HFrEF. In the early stages of pressure overload or cardiac injury, mitophagy is transiently activated and may help preserve mitochondrial quality (112, 113). As the disease advances, mitophagic flux may become insufficient or impaired. Damaged mitochondria then accumulate, increasing ROS production and further worsening cardiac function (74, 75, 112). Whether this adaptive increase in ketone utilization is causally linked to altered mitophagic flux in HFrEF remains unclear.

##### 6.2.1.2 HFpEF

HFpEF is a heterogeneous syndrome that includes cardiometabolic and non-cardiometabolic phenotypes with distinct metabolic features.

###### 6.2.1.2.1 Cardiometabolic HFpEF

Cardiometabolic HFpEF is commonly associated with obesity, type 2 diabetes, insulin resistance, hypertension, and chronic low-grade inflammation (114–116). Adipose tissue dysfunction increases circulating FFAs and promotes myocardial lipid accumulation and lipotoxicity (115, 117). Insulin-stimulated glucose oxidation is also impaired, which further reduces metabolic flexibility (118).

At the mitochondrial level, this phenotype is associated with nicotinamide adenine dinucleotide (NAD⁺) deficiency, increased protein acetylation, and oxidative stress (119). The mitophagic response to metabolic stress, particularly lipid overload, may also be blunted (119, 120). Experimental studies suggest that insufficient mitophagy promotes the accumulation of damaged mitochondria (120). This may increase oxidative stress, worsen endothelial dysfunction, and impair myocardial relaxation (120, 121). Ketone body metabolism in cardiometabolic HFpEF is less consistent than in HFrEF. Some studies report reduced myocardial ketone body uptake or oxidation, suggesting a limited ability to use ketone bodies as an alternative fuel (122). Other studies have observed increased cardiac HMGCS2 expression and proposed that local ketogenesis may support NAD⁺ balance during metabolic stress (123). However, the functional importance of this response remains uncertain. Whether blunted mitophagy contributes to altered ketone utilization in this phenotype also remains unclear.

###### 6.2.1.2.2 Non-cardiometabolic HFpEF

Non-cardiometabolic HFpEF includes phenotypes associated with aging, valvular heart disease, hypertrophic remodeling, atrial fibrillation, or amyloidosis (124–126). These phenotypes may show reduced oxidative reserve, mitochondrial dysfunction, and increased oxidative stress. Unlike cardiometabolic HFpEF, they are less closely linked to systemic metabolic syndrome. Their pathophysiology is more strongly characterized by structural remodeling, myocardial stiffness, fibrosis, and impaired ventricular-vascular coupling (38, 127). Direct evidence on ketone metabolism, mitophagy, and their interaction is sparse in these phenotypes. Any proposed link between the two processes therefore remains largely conceptual.

Overall, metabolic remodeling differs across heart failure phenotypes. Increased ketone body utilization is a relatively consistent adaptive feature of HFrEF and may partly compensate for impaired energy production. Cardiometabolic HFpEF is often associated with persistent reliance on FAO, reduced metabolic flexibility, and a blunted mitophagic response, whereas changes in ketone utilization remain inconsistent (128). The metabolic and mitochondrial features of non-cardiometabolic HFpEF are less clearly defined. These differences underscore the phenotype-dependent nature of metabolic remodeling in heart failure.

#### 6.2.2 Ischemic heart disease

Ischemic heart disease causes marked changes in myocardial energy metabolism because the heart depends heavily on a continuous oxygen supply. During ischemia, oxygen deprivation suppresses mitochondrial oxidative phosphorylation. It also limits the oxidation of FAs, glucose-derived pyruvate, and ketone bodies (129). The myocardium therefore increases its reliance on anaerobic glycolysis to maintain ATP production (130, 131). However, glycolysis generates relatively little ATP and promotes lactate and proton accumulation, leading to intracellular acidosis (131). Impaired mitochondrial oxidation also causes the accumulation of succinate and partially oxidized lipid intermediates (132).

Reperfusion rapidly restores oxygen and substrate delivery but can also aggravate mitochondrial injury. Accumulated succinate is rapidly oxidized and drives reverse electron transport at mitochondrial complex I (132). This process produces a burst of ROS (132). Together with calcium overload and mitochondrial permeability transition pore opening, excessive ROS promotes cardiomyocyte injury during reperfusion (133). Mitophagy contributes to mitochondrial quality control during ischemia-reperfusion injury. PINK1-PRKN, FUNDC1, and ULK1/Rab9-mediated pathways can remove damaged mitochondria and help maintain cellular homeostasis (134–136). This response may be particularly important during ischemia, when mitochondrial damage progressively accumulates. During reperfusion, however, mitophagic flux may become insufficient or disrupted. Damaged mitochondria may therefore persist and further increase oxidative stress and cell death (136, 137). The timing and extent of mitophagy are thus likely to influence its protective effects.

Ketone body metabolism also changes across different stages of ischemia-reperfusion injury. During acute ischemia, limited mitochondrial oxidative capacity suppresses myocardial ketone body oxidation (138). *β*-OHB may consequently accumulate in the myocardium or circulation (138, 139). By contrast, experimental administration of *β*-OHB at the onset of reperfusion has been associated with smaller infarct size, improved mitochondrial function, and enhanced autophagic flux (109, 140). These findings suggest that the effects of *β*-OHB may depend on oxygen availability, treatment timing, and mitochondrial metabolic capacity. However, they do not establish that endogenous ketone body accumulation during ischemia is directly harmful.

Whether *β*-OHB protects the reperfused myocardium through selective mitophagy, rather than broader autophagic, metabolic, or antioxidant mechanisms, remains unresolved.

#### 6.2.3 Hypertensive and hypertrophic cardiomyopathy

Hypertensive heart disease undergoes progressive metabolic remodeling from adaptive hypertrophy to decompensation (141). During the early stage, cardiomyocytes increase glucose uptake and glycolysis, which may temporarily improve oxygen efficiency (142). However, glycolysis is often poorly coupled to mitochondrial glucose oxidation. This mismatch promotes proton accumulation, intracellular acidosis, and impaired calcium handling, thereby reducing contractile efficiency (143, 144).

As the disease progresses, myocardial metabolism becomes more heterogeneous. FAO may initially remain unchanged or increase but often declines as mitochondrial dysfunction and oxidative stress develop (141, 145). Ketone body utilization may also vary with disease stage. When mitochondrial function is relatively preserved, greater ketone body availability could provide an alternative source of acetyl-CoA and support ATP production during metabolic stress (146). In advanced disease, mitochondrial dysfunction may reduce ketolytic capacity and limit the energetic contribution of ketone bodies (147). Mitophagy also changes during disease progression. Moderate activation during early hypertrophy may remove mitochondria damaged by mechanical stress and increased metabolic demand (148). Chronic pressure overload, however, can impair PINK1-PRKN-dependent and receptor-mediated mitophagy (143). Inadequate mitochondrial clearance may then promote the accumulation of dysfunctional mitochondria and further reduce oxidative capacity (143). These observations suggest that ketone body utilization and mitophagy may both vary with disease stage, but a direct relationship between them has not been tested.

Hypertrophic cardiomyopathy (HCM) has a metabolic profile distinct from hypertensive hypertrophy. It is often caused by sarcomeric mutations that increase energy demand and reduce mechanical efficiency (149). Cardiomyocytes therefore develop early energetic deficits and undergo metabolic reprogramming. Reported changes include reduced FAO and increased glycolysis (149). Reduced FAO may result from mitochondrial dysfunction or represent an adaptive attempt to improve oxygen efficiency (150, 151). Increased glycolysis can provide rapid ATP production but may not meet sustained energy demands when mitochondrial oxidative capacity is impaired (152).

Ketone body metabolism in HCM remains poorly characterized. Increased ketone body utilization could provide a compensatory fuel pathway during chronic energetic stress, but this possibility has not been directly demonstrated. Mitophagy may help preserve mitochondrial quality by removing damaged organelles, although its effects are likely context-dependent. Excessive mitochondrial clearance could further reduce oxidative capacity, whereas insufficient clearance may promote oxidative stress and metabolic inefficiency (153). In contrast, insufficient mitophagy may promote mitochondrial accumulation, oxidative stress, and metabolic inefficiency (154). A mitophagy-ketone interaction in HCM is therefore plausible, but remains unproven.

#### 6.2.4 Age-Related myocardial degeneration

Age-related myocardial degeneration is accompanied by gradual metabolic remodeling and impaired mitochondrial quality control. Mitophagy participates in this process, but its activity may change across aging stages (155, 156). Unlike acute cardiac stress, aging causes slow and cumulative changes in substrate utilization, mitochondrial turnover, and intracellular signaling (157, 158). The aged myocardium generally shows reduced metabolic flexibility. Its ability to switch among FAs, glucose, and ketone bodies may become impaired. FAO can decline because of reduced mitochondrial density and defects in *β*-oxidation enzymes (159). Glucose oxidation may also become poorly coupled to glycolysis, resulting in less efficient ATP production (160).

Changes in cardiac ketone body metabolism during aging remain incompletely defined. Circulating ketone body levels may increase because of systemic metabolic changes, whereas myocardial ketone body utilization may not increase proportionally (161). Reduced ketolytic enzyme activity, impaired TCA cycle flux, and electron transport chain dysfunction could limit ketone oxidation (162). These changes may create a mismatch between substrate availability and mitochondrial oxidative capacity. However, direct evidence for impaired ketolysis in the aged human heart remains limited. Beyond energy provision, ketone bodies may exert signaling effects in the aged myocardium. *β*-OHB can regulate inflammation, oxidative stress, and gene expression. Proposed mechanisms include histone deacetylase inhibition and modulation of redox-sensitive transcription factors (105, 163). These mechanisms may contribute to stress adaptation, but their direct impact on cardiac aging remains uncertain. Aging is also associated with defects at several stages of mitophagy. These may include reduced autophagosome formation, altered expression of mitophagy-related proteins, impaired recognition of damaged mitochondria, and defective autophagosome–lysosome fusion (164). In addition, lysosomal dysfunction may further reduce mitophagic flux by limiting acidification and enzymatic activity (165). As a result, dysfunctional mitochondria accumulate, promoting oxidative stress, mitochondrial DNA damage, and calcium imbalance (82, 83).

During cardiac aging, reduced substrate oxidation may aggravate mitochondrial stress and increase the demand for mitochondrial clearance (166). Conversely, inadequate mitophagy may allow mitochondria with poor oxidative capacity to accumulate, further limiting the utilization of available substrates, including ketone bodies (167). This could create a self-reinforcing cycle of mitochondrial dysfunction and metabolic inefficiency. However, whether impaired mitophagy directly limits ketone oxidation, or ketone signaling modifies mitophagy in the aged heart, remains unknown.

#### 6.2.5 Atrial fibrillation-associated metabolic remodeling

Atrial fibrillation (AF) is associated with significant metabolic remodeling in atrial cardiomyocytes. These changes involve several pathways and may reflect both adaptive and maladaptive responses to sustained electrical and mechanical stress. Reduced mitochondrial oxidative phosphorylation is commonly observed, often together with lower FAO and greater reliance on glycolysis (168, 169). Changes in amino acid metabolism, lipid handling, and nucleotide turnover have also been reported (170, 171). These shifts may initially help maintain ATP production during rapid atrial activation. Over time, however, they may become insufficient and contribute to energy imbalance, oxidative stress, calcium dysregulation, and structural remodeling (172).

The role of ketone body metabolism in AF remains incompletely understood. Higher levels of *β*-OHB and ketogenic amino acid metabolites have been detected in atrial tissue from patients with persistent AF (168). These findings may reflect a response to impaired mitochondrial energy metabolism, but they do not establish causality. Results also differ across clinical settings. In aging-related AF, lower *β*-OHB levels have been associated with reduced mitochondrial enzyme activity, impaired oxidative capacity, and more severe atrial remodeling (173). Population studies show a U-shaped relationship between circulating ketone bodies and AF risk (174), although these observational findings cannot establish causation. Experimental studies further suggest that prolonged or high *β*-OHB exposure may reduce mitochondrial biogenesis, increase cardiomyocyte apoptosis, and promote atrial fibrosis (175). The effects of ketone bodies may therefore depend on concentration, exposure duration, and metabolic context.

Altered mitochondrial quality control has also been reported in AF. Human atrial tissue and experimental models show abnormal mitochondrial accumulation, altered mitophagy-related structures, and reduced mitochondrial clearance (176, 177). Reduced mitophagy may allow damaged mitochondria to persist, increasing ROS production, lowering mitochondrial membrane potential, and impairing ATP generation (176). These abnormalities may contribute to electrical instability, defective calcium handling, and progressive atrial remodeling. Whether *β*-OHB directly modulates atrial mitophagy, or whether mitophagic dysfunction alters ketone utilization, remains unknown.

## 7 Clinical translation of mitophagy and ketone body metabolism

The metabolic phenotypic differences mentioned above, especially the disease-specific alterations in ketone body metabolism and mitophagy, provide a potential entry point for clinical translation. However, the extent to which these mechanistic findings can genuinely guide clinical decision-making, making depends on their applicability to diagnostic stratification and therapeutic intervention. This section explores the pathways for clinical translation and the current limitations regarding these findings, focusing on three key aspects: biomarkers, intervention strategies, and precision therapy.

### 7.1 Biomarkers

Clinical translation of mitophagy and ketone body requires biomarkers that can be repeatedly measured and interpreted across cardiac phenotypes. Candidate markers include circulating ketone bodies, breath metabolites, plasma metabolomic profiles, indices of myocardial energetics, and mitochondrial or mitophagy-related indicators. These markers reflect different levels of metabolic regulation. Circulating metabolites mainly indicate systemic substrate availability and metabolic stress, whereas imaging and myocardial tissue measurements more directly reflect cardiac energetics and mitochondrial quality control.

#### 7.1.1 Circulating ketone bodies and breath acetone

Circulating *β*-OHB, ACAC, and total ketone bodies have been studied as markers of HF severity and prognosis (178). Elevated *β*-OHB levels are associated with higher natriuretic peptides, impaired right ventricular function, and neurohormonal activation in advanced HFrEF (179). However, this association weakens after adjustment for FFAs, suggesting that *β*-OHB partly reflects systemic lipolysis and metabolic stress rather than independently predicting outcomes (179). The ratio between *β*-OHB and ACAC may provide additional information. The *β*-OHB to ACAC ratio reflects redox balance through the BDH1-mediated conversion linked to the NADH/NAD⁺ system (180). A higher *β*-OHB/ACAC ratio generally indicates a more reduced mitochondrial environment (180). An early clinical study found that a low arterial ACAC/*β*-OHB ratio predicted mortality in acute HF (181). Nevertheless, circulating ketone bodies are produced mainly in the liver. The blood ketone body ratio therefore reflects hepatic and systemic redox conditions more directly than myocardial redox status.

Breath AC offers a noninvasive method for assessing ketone-related metabolic changes. AC is generated by the spontaneous decarboxylation of ACAC and can be detected in exhaled air (182). Breath AC is higher in HF than in healthy controls and increases during acute decompensation (183). In chronic HF, high breath AC was associated with cardiac and all-cause mortality after adjustment for conventional clinical variables (184). However, breath AC is influenced by fasting, diet, diabetes, exercise, sampling procedures, and analytical platforms. A meta-analysis identified substantial between-study heterogeneity (185). Standardized sampling and validated thresholds are therefore required before breath AC can be used routinely.

#### 7.1.2 Tissue and circulating indicators of mitophagy

The clinical assessment of cardiac mitophagy remains difficult. Common markers include PINK1, PRKN, phosphorylated ubiquitin, BNIP3, FUNDC1, LC3, and p62. Analyses of failing human hearts have shown reduced expression of multiple mitophagy-related genes in advanced HF, including PINK1, PRKN, FUNDC1, BNIP3, and beclin 1 (BECN1) (186). These markers cannot be interpreted in isolation. PINK1 accumulation may indicate mitochondrial damage, whereas reduced PINK1 or PRKN may indicate limited pathway capacity. An increase in LC3 may reflect greater autophagosome formation, but it may also result from impaired lysosomal degradation. Similarly, p62 accumulation may indicate defective substrate clearance, although p62 is also regulated at the transcriptional level. Additional evidence can be obtained by measuring PRKN translocation to mitochondria, colocalization between mitochondrial proteins and LC3 or lysosomal markers, and the presence of mitochondria within autophagosomes or autolysosomes. These approaches are more specific than total protein abundance. However, they require myocardial tissue and remain largely research methods. Endomyocardial biopsy also has sampling limitations and cannot be used for frequent monitoring.

Peripheral blood may offer a less invasive alternative. Patients with chronic HF show altered mitochondrial structure, increased mitochondrial reactive oxygen species, reduced mitochondrial mass, and changes in mitophagic flux in circulating leukocytes (187). Such measurements could support dynamic monitoring of systemic mitochondrial responses. However, immune-cell mitophagy is affected by inflammation, infection, diabetes, kidney disease, and medication. It cannot be assumed to represent cardiomyocyte mitophagy directly.

#### 7.1.3 An integrated biomarker strategy

A practical diagnostic framework may combine several complementary levels. Circulating ketone bodies and breath AC could reflect systemic ketone metabolism and disease severity. Tissue or peripheral blood cell panels could provide information about mitophagy-related signaling. This integrated approach may be more useful than relying on one biomarker. Future prospective studies should determine whether these panels improve diagnosis, prognosis, and treatment selection beyond established clinical tools.

### 7.2 Effects of cardiovascular therapies on mitophagy and ketone body metabolism

Growing evidence suggests that several cardiovascular therapies influence cardiac function partly by modifying mitochondrial quality control or metabolic substrate utilization. These effects may involve the restoration of mitophagy, increased ketone body availability, enhanced myocardial ketone oxidation, or coordinated changes in both processes. However, the strength of evidence differs substantially among interventions. Table 2 summarizes the major interventions discussed in this section, with emphasis on their reported effects on ketone body metabolism and mitophagy, potential target populations, major adverse effects.

**Table 2:** Effects of cardiovascular therapies on ketone body metabolism and mitophagy.

Intervention	Effect on ketone body metabolism	Effect on mitophagy	Potential target population	Main adverse effects or limitations
Exogenous ketone body supplementation	Directly increases circulating ketones and myocardial ketone availability	Possible indirect modulation; not demonstrated in human myocardium	Patients with HFrEF or HFpEF	Gastrointestinal discomfort, electrolyte load, excessive ketosis; long-term safety unclear
SGLT2 inhibitors	Modestly increase circulating ketone bodies and may enhance ketone body oxidation	Activate FUNDC1 or PINK1-PRKN- mediated mitophagy in experimental models	HFrEF, HFpEF, type 2 diabetes, chronic kidney disease	Genital infections, volume depletion, hypotension, rare ketoacidosis
Sacubitril/valsartan	No established direct effect	May enhance mitophagy and mitochondrial function	Patients with HFrEF; selected HFpEF patients	Hypotension, hyperkalemia, renal dysfunction, angioedema
Statins	No established direct effect	May activate PINK1-dependent mitophagy	Atherosclerotic cardiovascular disease or high risk patients	Myalgia, liver enzyme elevation, rare rhabdomyolysis
PR-364	No established effect	Directly activates PRKN-mediated mitophagy	Potentially patients after myocardial infarction	Human safety, dose, and therapeutic window unknown
Urolithin A	No established direct effect	Activates AMPK-mTOR- associated mitophagy	Potentially HFpEF or mitochondrial dysfunction phenotypes	Generally mild gastrointestinal effects; efficacy uncertain
Exercise training	May improve metabolic flexibility and ketone utilization indirectly	May activate PINK1-PRKN- mediated mitophagy	Stable HF, post-MI, or cardiac rehabilitation populations	Exercise-related injury or ischemic symptoms in poorly selected patients
Melatonin	No established direct effect	May restore insufficient mitophagy or suppress excessive mitophagy, depending on context	Potentially ischemia-reperfusion injury; investigational use	Somnolence, dizziness, drug interactions; optimal timing unclear
Ketogenic diet	Increases hepatic ketogenesis and circulating ketone bodies; effects on cardiac ketone body oxidation vary	Direct effect remains unclear	Selected metabolic phenotypes under close supervision	Dyslipidemia, gastrointestinal symptoms, nutrient deficiencies, hypoglycemia or ketoacidosis risk

“Potential target population” does not represent an approved indication for experimental interventions.

#### 7.2.1 Exogenous ketone body supplementation

Exogenous ketone body supplementation provides the most direct approach to increasing circulating ketone body availability. In patients with chronic HFrEF, intravenous infusion of *β*-OHB increased cardiac output and left ventricular ejection fraction without causing a major increase in blood pressure (97). A subsequent randomized, double-blind crossover trial showed that 14 days of oral ketone ester treatment increased resting cardiac output and reduced cardiac filling pressures and ventricular volumes in patients with HFrEF receiving standard medical therapy (188). Similar hemodynamic effects were reported in patients with type 2 diabetes and HFpEF, in whom two weeks of ketone ester treatment increased cardiac output and reduced pulmonary capillary wedge pressure, particularly during exercise (189). These findings indicate that the failing human heart retains the capacity to respond to an increased ketone body supply. Ketone bodies may also increase cardiac contractility, reduce systemic vascular resistance, alter myocardial blood flow, and act as signaling metabolites. Moreover, an acute crossover trial in symptomatic HFpEF showed that ketone ester administration reduced indices of cardiac filling pressure and carbohydrate utilization but did not improve peak oxygen consumption or exercise duration (190). Thus, short-term hemodynamic improvement does not necessarily translate into improved exercise capacity or long-term clinical outcomes. None of these human trials directly measured myocardial mitophagy. It therefore remains unclear whether exogenous ketones modify mitochondrial turnover in patients or whether their effects primarily result from metabolic and vascular mechanisms.

#### 7.2.2 Sodium-glucose cotransporter 2 inhibitors

Sodium-glucose cotransporter 2 (SGLT2) inhibitors are particularly relevant to the interaction between mitophagy and ketone body metabolism. These drugs can modestly increase circulating ketone body concentrations by promoting glucosuria, reducing the insulin to glucagon ratio, and increasing hepatic FAO and ketogenesis (191). Experimental studies also indicate that they may increase myocardial ketone utilization and restore mitochondrial quality control. However, these effects are unlikely to represent a single unified mechanism and may vary according to the drug, disease model, and stage of cardiac injury.

In cardiac microvascular ischemia-reperfusion models, empagliflozin activated the AMPK*α*1-ULK1-FUNDC1 pathway, thereby enhancing FUNDC1-dependent mitophagy, preserving mitochondrial function, and reducing microvascular injury (192). Dapagliflozin similarly attenuated myocardial ischemia-reperfusion injury by activating AMPK and the PINK1-PRKN pathway (193). In diabetic cardiomyopathy, canagliflozin promoted PINK1-PRKN-mediated mitophagy and improved mitochondrial function (194). Silencing PINK1 weakened these effects, providing evidence that mitophagy contributed directly to the observed cardioprotection (195). These studies suggest that SGLT2 inhibitors may activate both receptor-mediated and ubiquitin-dependent mitophagy pathways, depending on the pathological setting.

SGLT2 inhibitors may also influence cardiac ketone body metabolism. In isolated rat hearts exposed to ischemia and reperfusion, empagliflozin shifted substrate utilization away from glucose oxidation and toward ketone body utilization, accompanied by improved ATP and phosphocreatine levels and better functional recovery (196). More direct causal evidence was obtained in a murine post-infarction HF model. Empagliflozin improved cardiac contractility in control mice, whereas this effect was attenuated in mice with cardiomyocyte-specific deletion of BDH1, which limits myocardial *β*-OHB oxidation (197). In diabetic cardiomyopathy models, empagliflozin also increased the activity or expression of the ketolytic enzymes BDH1 and SCOT, while reducing oxidative stress and mitochondrial damage (96).

These findings support the possibility that SGLT2 inhibitors improve mitochondrial fitness through parallel effects on ketone body oxidation and mitophagy. Increased ketone body oxidation may provide an additional oxidative substrate and alter the mitochondrial redox state, whereas mitophagy may preserve the mitochondrial population required for efficient ketolysis. However, these two effects have rarely been assessed simultaneously in the same experimental system. More importantly, no human study has yet demonstrated that the cardiovascular benefits of SGLT2 inhibition depend on increased myocardial mitophagy or ketone oxidation. In addition, therapeutic ketosis induced by SGLT2 inhibitors must be distinguished from pathological ketoacidosis, particularly during fasting, acute illness, insulin deficiency, or volume depletion.

#### 7.2.3 Cardiovascular therapies and mitophagy

Several established cardiovascular treatments may affect mitophagy through mechanisms that are not part of their primary pharmacological actions.

Sacubitril/valsartan may enhance mitochondrial quality control by increasing biologically active atrial natriuretic peptide through neprilysin inhibition. In peripheral blood mononuclear cells obtained from a small group of patients with HFrEF, two months of sacubitril/valsartan treatment was associated with increased atrial natriuretic peptide levels, enhanced autophagy- and mitophagy-related responses, improved mitochondrial membrane potential, and reduced reactive oxygen species production (198). However, these findings were obtained from circulating cells rather than myocardial tissue, and the sample size was small. They should therefore be considered preliminary evidence rather than direct confirmation of restored mitophagy in the human heart.

Statins may also regulate mitophagy. Acute simvastatin treatment suppressed AKT-mechanistic target of rapamycin (mTOR) signaling and activated PINK1-dependent mitophagy in cardiomyocytes and mouse hearts subjected to ischemia-reperfusion injury (199). The loss of protection in PRKN-deficient animals suggested that mitophagy was required for the acute cardioprotective effect (199). In an atherosclerosis model, pitavastatin activated a calcium-dependent protein kinase I (CAMK1)-PINK1-PRKN pathway in endothelial progenitor cells, thereby restoring mitophagic flux, supporting cell proliferation, and promoting vascular repair (200). These findings suggest that statins may influence mitochondrial quality control in both cardiac and vascular cells. Nevertheless, it remains uncertain whether these mechanisms contribute substantially to the established clinical benefits of long-term statin therapy.

#### 7.2.4 Direct interventions targeting mitophagy

More selective mitophagy-targeted compounds are being developed, although their cardiovascular application remains predominantly preclinical. PR-364 is a small-molecule PRKN activator that increased PRKN-dependent mitophagy and mitochondrial biogenesis after myocardial infarction. Administration after coronary occlusion reduced adverse left ventricular remodeling and improved mitochondrial ATP production in experimental models (201). This study provides proof of principle that direct pharmacological activation of PRKN may be therapeutically useful after myocardial injury. However, the appropriate dose, therapeutic window, tissue specificity, and long-term safety of PRKN activation have not yet been established in humans.

Urolithin A, a gut microbiota-derived metabolite and mitophagy inducer, has also been investigated. In a recent preclinical HFpEF study, urolithin A activated mitophagy through the AMPK-mTOR axis and altered the gut microbiota-ceramide pathway, resulting in reduced cardiac remodeling and improved cardiac function (202). In contrast, a small randomized crossover trial involving ten patients with HFrEF found that four weeks of urolithin A treatment did not significantly improve left ventricular ejection fraction, ventricular volumes, right ventricular function, pro-B-type natriuretic peptide, or inflammatory markers (203). The difference between the experimental and clinical results highlights the translational gap between the activation of mitophagy in controlled models and clinically meaningful improvements in patients with established HF.

#### 7.2.5 Exercise and regulation of mitophagy

Exercise training represents a nonpharmacological intervention that may improve mitochondrial quality control. In mice with myocardial infarction, resistance exercise increased irisin and fibronectin type III domain-containing protein 5 signaling, activated the PINK1-PRKN pathway, improved myocardial mitophagy, and reduced oxidative stress and cardiac dysfunction (204). Although these findings provide a mechanistic explanation for some benefits of exercise-based cardiac rehabilitation, evidence that exercise restores mitophagic flux in the human myocardium remains limited.

The therapeutic objective should not always be to maximize mitophagy. Moderate mitophagy removes dysfunctional mitochondria and limits oxidative stress, whereas insufficient mitophagy permits damaged mitochondria to accumulate and excessive or prolonged mitochondrial clearance may reduce mitochondrial mass and impair energy production. This context dependence is illustrated by studies of melatonin in myocardial ischemia-reperfusion injury. Melatonin has been reported to restore impaired OPA1-related mitochondrial fusion and mitophagy through AMPK signaling in some models (205), whereas other studies suggest that it protects the myocardium by suppressing excessive mitophagy through sirtuin 3 (SIRT3)-related pathways (206). These apparently opposing observations indicate that the direction of an effective intervention depends on the baseline mitophagic state, the timing of administration, and the severity of mitochondrial injury.

#### 7.2.6 Ketogenic diets

Ketogenic diets should be distinguished from exogenous ketone supplementation. Exogenous ketone esters primarily increase circulating ketone concentrations, whereas ketogenic diets simultaneously alter FA availability, glucose oxidation, insulin signaling, amino acid metabolism, and hepatic ketogenesis. Their cardiovascular effects therefore cannot be attributed to ketone bodies alone. In mice with cardiac mitochondrial pyruvate carrier deficiency, a ketogenic diet improved HF even when cardiomyocyte ketone oxidation was genetically impaired, indicating that ketone oxidation was not required for the therapeutic effect (207). In contrast, a ketogenic diet did not improve cardiac function in a mouse model of ischemic HF. It increased reliance on FAO, suppressed insulin-stimulated glucose oxidation, and reduced cardiac mechanical efficiency (208). These findings demonstrate that nutritional ketosis may produce disease-specific metabolic effects and should not be considered equivalent to pharmacological ketone delivery.

#### 7.2.7 Current limitations

Collectively, current evidence indicates that cardiovascular therapies can modulate mitophagy and ketone body metabolism through multiple, partially overlapping mechanisms. However, these interventions differ substantially in their levels of evidence, and most mechanistic findings remain preclinical or derived from small translational studies. Several important limitations currently restrict clinical translation. First, a major gap exists between experimental models and human disease. Most direct evidence originates from cellular or animal studies, whereas human trials have largely focused on hemodynamic or metabolic endpoints without direct assessment of myocardial mitophagic flux. Second, causality remains uncertain. For example, increases in circulating ketone bodies do not necessarily reflect enhanced myocardial ketone oxidation, and improved cardiac function observed with SGLT2 inhibitors or ketone body supplementation cannot yet be attributed definitively to changes in mitochondrial quality control. Third, methodological limitations hinder mechanistic interpretation. Reliable *in vivo* quantification of mitophagy in the human heart is still lacking, and peripheral biomarkers may not accurately reflect myocardial biology. Similarly, integrated assessment of ketone metabolism, mitochondrial turnover, and cardiac energetics within the same experimental or clinical framework is rarely performed, making it difficult to determine whether these processes act independently or synergistically. Fourth, therapeutic effects appear to be highly context-dependent. Both insufficient and excessive mitophagy may be detrimental, depending on disease stage, metabolic state, and timing of intervention. These observations highlight the need to define patient subgroups. Finally, safety, dosing, and long-term outcomes remain incompletely characterized for emerging therapies.

Future research should therefore move toward integrated, mechanism-based clinical phenotyping. This includes simultaneous evaluation of myocardial energetics, ketone body oxidation, and mitophagic flux using advanced techniques. Large-scale, long-term clinical trials are also required to determine whether modulation of these pathways translates into meaningful improvements in symptoms, functional capacity, hospitalization rates, and survival. Ultimately, a precision medicine approach that stratifies patients according to mitochondrial function and metabolic profile may be necessary to fully exploit the therapeutic potential of targeting mitophagy and ketone body metabolism in cardiovascular disease.

## 8 Conclusion

Mitophagy and ketone body metabolism are connected through mitochondrial function and cardiac metabolic adaptation. Mitophagy may preserve the mitochondrial capacity required for ketone oxidation. Conversely, ketone bodies, particularly *β*-OHB, may influence mitophagy through metabolic and signaling pathways. Current evidence more strongly supports ketone body-mediated regulation of mitophagy than the reverse direction. However, this difference may partly reflect the greater focus of previous interventional studies on ketone body-based treatments. Direct evidence that mitophagic flux controls myocardial ketone oxidation remains limited, and the relationship may differ across disease phenotypes and stages.

Future studies should determine whether mitophagy directly affects myocardial ketone oxidation, ATP production, and cardiac energetics. These processes should be measured in the same experimental models to establish causality. Studies should also identify the disease phenotypes and stages most likely to benefit from targeting this interaction. Comparisons among HFrEF, cardiometabolic HFpEF, and ischemia–reperfusion injury will be particularly informative. In addition, the metabolic and signaling effects of *β*-OHB should be distinguished. Ketolysis-deficient models, receptor-specific interventions, and pathway-selective inhibitors may help clarify whether *β*-OHB regulates mitophagy through oxidation-dependent or independent mechanisms. The safety and context of these interventions also require careful consideration. Both insufficient and excessive mitochondrial clearance may be harmful, depending on baseline mitochondrial function and disease stage. Ketone body supplementation and ketogenic diets should be evaluated separately because they produce different systemic metabolic effects. Clinical trials should combine conventional cardiovascular outcomes with measures of ketone utilization, mitochondrial function, and mitophagy-related target engagement.

Overall, the proposed mitophagy-ketone body interaction offers a useful framework for understanding cardiac metabolic adaptation. Its therapeutic relevance will depend on stronger causal evidence, appropriate patient selection, and clinical studies that connect pathway modulation with meaningful cardiovascular outcomes.
